# Biases in α-synuclein immuno-quantitation: a core problem for basic and ancillary studies of Parkinson’s disease and multiple system atrophy

**DOI:** 10.1186/s40035-024-00408-x

**Published:** 2024-03-25

**Authors:** Florent Laferrière, Ludivine Sabatier, Stéphane Claverol, Francesca De Giorgi, François Ichas

**Affiliations:** 1 Univ. Bordeaux, CNRS, IMN, UMR 5293, 33000 Bordeaux, France; 2 Bordeaux Proteome, Univ. Bordeaux, Bordeaux, France; 3 DiSTeBA, Univ. Salento, Anatomia Umana, Lecce, Italy

Parkinson’s disease (PD) and multiple system atrophy (MSA) are distinct neurodegenerative disorders sharing the accumulation of pathological alpha-synuclein (α-syn) amyloids in neurons or glial cells. Amyloidogenesis arises with the nucleation of β-sheet folded α-syn assemblies serving as conformational templates in the amyloid buildup at the expense of monomeric α-syn (for review see [1]). This pathological aggregation process can be experimentally mimicked in test tubes with recombinant α-syn [2], or by treatment with brain-derived α-syn amyloids or synthetic α-syn pre-formed fibrils (PFFs) in biological systems [3–5]. Noteworthy, scoring pathological aggregation in biological samples and qualifying the specific seeding propensity of extracts for comparing them both depend on the capability of identifying and quantifying the amyloid share of total α-syn.

Here, we used samples from control, PD and MSA subjects, and sought to compare their respective amyloid load after routine homogenization and addition of sarkosyl. We observed that the amyloid forms were not resolved by SDS-PAGE, although this method is frequently used for the analysis of pathological α-syn. Indeed, the amyloid assemblies were mostly retained in the stacking gel and partially disassembled to an unpredictable extent by the denaturing conditions, especially by SDS (Fig. 1a, Additional file 1: Fig. S1a).

**Fig. 1:**
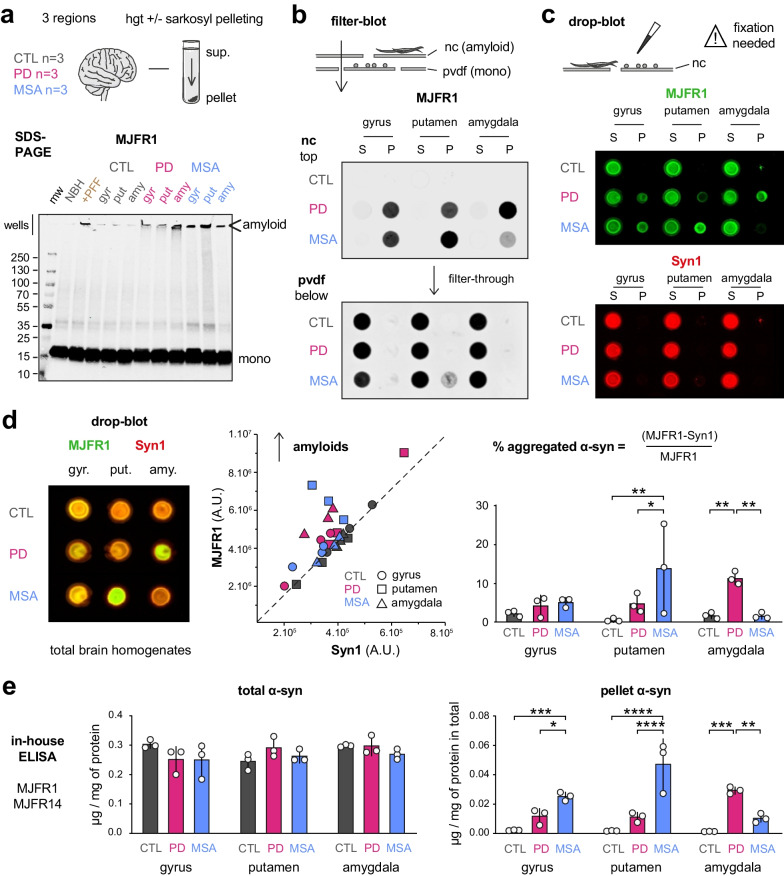
Analysis of distinct α-syn species derived from homogenates or sarkosyl-pelleting fractions of three distinct brain regions (cingulate gyrus, putamen, amygdala) from *n* = 3 independent control, PD and MSA subjects, respectively. **a** SDS-PAGE analysis of total α-syn (MJFR1) depicts a monomeric band while amyloids are retained in the wells. **b** Filter-blot analysis of total (MJFR1) α-syn retained on the top nitrocellulose and the underneath PVDF membranes in the supernatant (S) and pellet (P) sarkosyl-pelleting fractions. **c** Drop-blot analysis of total (MJFR1, green) and non-amyloid (Syn1, red) α-syn in the same fractions. Samples were fixed with 4% PFA after dropping. **d** Left: same drop-blot analysis on total brain homogenates before pelleting. Middle: samples are plotted with their respective Syn1 (x-axis) and MJFR1 (y-axis) signal intensities. Right: proportion of aggregated α-syn calculated as indicated (mean ± SD). **e** α-Syn amounts measured with our in-house ELISA (MJFR1 capture, MJFR14 detection) in total brain homogenates (left) and sarkosyl-insoluble pellets (right) as µg of α-syn per mg of protein in total brain homogenates (mean ± SD). Significant differences obtained from Tukey-corrected two-way ANOVAs are represented (*P < 0.05; **P < 0.005; ***P < 0.0005; ****P < 0.0001)

We thus submitted brain homogenate samples to non-denaturing (native) analysis procedures. Filter-blot is based on the principle that aggregated proteins are preferentially retained on nitrocellulose (NC) membranes while monomers can flow-through and are lost, or alternatively retained on an underneath PVDF membrane [6]. The sarkosyl-insoluble component of the brain homogenates was pelleted by ultracentrifugation and two resulting fractions (supernatant, pellet) were submitted to filter-blot (Fig. 1b, Additional file 1: Fig. S1b). At variance from control, MJFR1 immunoblotting showed that a fraction of PD and MSA brain-derived α-syn was specifically retained on the NC membrane, corresponding to α-syn aggregates. This was confirmed in parallel by immunoblot experiments revealed with an anti-pS129 α-syn antibody (EP1536Y) (Additional file 1: Fig. S1b). By using the antibody Syn1, which is specific for non-amyloid α-syn [7], we confirmed that the non-amyloid α-syn was predominantly present in supernatants, crossed the NC membrane and was eventually detected on the underneath PVDF membrane. We have also confirmed that using the MJFR1-Syn1 immunolabelling pair allows, by deduction, to identify amyloid α-syn [7]. These data indicate that unlike SDS-PAGE, filter-blots—especially when a single NC membrane is used—allow a robust inter-sample comparison of α-syn amyloid loads, inducing a physical separation of the different α-syn entities.

In order to compare the relative proportions of amyloid α-syn among all the α-syn species present in synucleinopathy brain samples, we thus sought to avoid any loss and/or physical separation of the different species and developed a simple drop-blot procedure. As previously reported, we found that in these conditions, monomeric α-syn needs to be fixed to the membrane with 4% PFA to prevent its unpredictable wash-out during processing, while amyloid assemblies remain firmly attached even without fixation [8] (Additional file 1: Fig. S2a). Drop-blots of supernatant/pellet fractions revealed with MJFR1 and Syn1 after 4% PFA fixation showed that non-amyloid (MJFR1- and Syn1-positive, supernatants) and amyloid (MJFR1-positive and Syn1-negative, pellets for PD and MSA) α-syn were both present on the same membrane. This approach allowed the direct identification of both soluble and insoluble α-syn species in brain homogenates without a separation step. Simultaneous quantification of Syn1 and MJFR1 signal intensities provided a “ratiometric” readout of the fraction of α-syn engaged in amyloid assemblies (Fig. 1d), well discriminating synucleinopathy vs control homogenates, and identifying brain regions with a stronger α-syn deposition: amygdala for PD and putamen for MSA. As mentioned, the signal ratio (MJFR1 − Syn1)/MJFR1 provides a direct readout of the percentage of amyloid vs total α-syn, ranging from 5% to 25% of amyloid α-syn in PD and MSA samples with a particularly high load in the putamen for MSA and in the amygdala for PD. In summary, we developed a fast and robust method to easily quantify the proportion of α-syn engaged into amyloid assemblies in human brain samples.

It is however of utmost importance to determine the absolute concentration of these amyloids, since this value directly impinges on disease spread and progression rates as well as on the possible subsequent seeding assays. Strikingly, we found that two commercial kits frequently used in the literature underestimate the amyloid fraction (Additional file 1: Fig. S3c), probably because they are using antibodies directed towards α-syn epitopes that are no longer accessible when α-syn is engaged into amyloid fibrils. This is a concern when analyzing amyloid-containing samples. We thus developed an in-house ELISA using two commercial α-syn antibodies detecting α-syn, independently of its engagement into amyloid assemblies: MJFR1 and MJFR14 for capture and detection, respectively (Fig. 1e, Additional file 1: Fig. S3) [7]. Indeed, unlike the routine commercial kits, our in-house ELISA achieved a relevant quantification of α-syn PFFs (Additional file 1: Fig. S3b, c). Using the in-house ELISA method for α-syn quantification in brain sample homogenates, we found that total α-syn concentrations were comparable among brain regions and patient groups, i.e., 0.2 to 0.3 µg/mg total protein. Insoluble amyloid α-syn was then pelleted and quantified as µg per mg of total protein determined before pelleting. This quantification again indicated a prominent α-syn pathology in the putamen of MSA (0.05 µg/mg) and the amygdala of PD patients (0.035 µg/mg of total protein). Additionally, these pelleted and total α-syn amounts measured by ELISA were unbiasedly validated by correlation with proteomic analysis of α-syn peptides in the respective samples (Additional file 1: Fig. S3d, Table S1). Being able to quantify all forms of α-syn in the different samples, we determined that the fraction of α-syn engaged into amyloid assemblies represented an average of 4.5% in the cingulate gyrus, 2.7% in the putamen and 10% in the amygdala for PD, and 10.5%, 18% and 4% for the corresponding regions in MSA (Additional file 1: Fig. S3e). Additionally, our measures of the percentage of aggregated α-syn by drop-blot, and quantitation of pelleted α-syn by ELISA, significantly correlated with both neuropathology scores of pathological inclusions and neuronal loss in the respective brain regions (Additional file 1: Fig. S4).

In summary, several pitfalls are encountered in the analysis of brain-derived α-syn. Even though western blotting remains a widespread method to characterize this type of samples [5], amyloid α-syn assemblies are not resolved by SDS-PAGE and unpredictably disassembled by denaturation with SDS. During native immunoblotting, amyloids are preferentially retained on the membrane compared to α-syn monomers that tend to be lost, leading to the likely overestimation of the amyloid fraction compared to monomers. This differential retention bias of different α-syn forms on the membrane is sometimes combined with antibody recognition problems which are generally ignored. For instance, in amyloid assemblies, the epitopes present in the non-amyloid-β component domain get masked, which prevents binding of antibodies directed to this region like the classical Syn1 (clone 42), making them “amyloid blind”. We observed that two ELISA kits routinely used in the literature, in which the antibodies used for capture and detection are not disclosed, blatantly failed to detect amyloid α-syn assemblies. In all likelihood, this was at the origin of severe misestimations of the α-syn contents of test samples, and certainly biased the conclusions regarding a possible conformational specificity of several antibodies targeting amyloid assemblies [9], or regarding the determination of their seeding activity per mass unit of protein [10].

In this study, we showed that the filter-blot allows to get a fast estimation of inter-sample amyloid load differences, while it overlooks the amounts of other α-syn forms. For instance, monomeric α-syn is extensively washed-out and lost from the membranes and needs to be maintained through an additional chemical fixation step to be reasonably quantified [8]. At the opposite, amyloid α-syn is well trapped on the membrane without fixation, however, it is often analyzed with Syn1, which is properly “amyloid-blind” [7]. We however decided to take advantage of this feature and developed a simple but robust drop-blot method for the quantification of relative proportions of amyloid α-syn among all the protein species in synucleinopathy brain samples. In addition, using a specific antibody pair in an in-house ELISA procedure, we came up with a user-friendly method to directly quantify total α-syn, irrespective of its assembly status, in brain homogenates and their subsequent fractions. This type of tool is required for the quantification of α-syn amyloids before using them as seeds in bioactivity assays, and we show how it can put a precise value on the α-syn amyloid load of brain samples. Even if the present study involves a relatively small number of patient samples, our work provides new tools and procedures with clear-cut results which can be extended in the future to other biological materials and functional analyses.

Collectively, this work highlights upstream quantification pitfalls skewing all the downstream considerations that can be made on the scoring and on the comparative seeding activity of clinical samples or fractions containing α-syn. We propose novel simple tools to avoid these pitfalls.

### Supplementary Information

**Additional file 1**. **Fig. S1**. SDS-PAGE and filter-blot of brain homogenate samples and sarkosyl-pelleting fractions. **Fig. S2**. Drop-blot of total brain homogenate samples and sarkosyl-pelleting fractions. **Fig. S3**. Development and validation of the in-house α-syn sandwich ELISA procedure. **Fig. S4**. Correlations of patients’ neuropathology scores with α-syn aggregation measurements. **Table S1**. Proteomic data summary of brain homogenates and sarkosyl-insoluble fractions. Materials and methods.

## Data Availability

The data used and/or analyzed in the current study are available from the corresponding author on reasonable request.

## Abbreviations

α-syn: Alpha-synuclein
PD: Parkinson’s disease
MSA: Multiple system atrophy
PFF: Pre-formed fibrils
