# Supplementary material for: Biases in α-synuclein immuno-quantitation: a core problem for basic and ancillary studies of Parkinson’s disease and multiple system atrophy
Source: Transl Neurodegener. 2024 Mar 25;13:15. doi: 10.1186/s40035-024-00408-x (PMC10962071; doi:10.1186/s40035-024-00408-x)
Supplement: Supplementary file 1 — Additional file 1. Fig. S1. SDS-PAGE and filter-blot of brain homogenate samples and sarkosyl-pelleting fractions. Fig. S2. Drop-blot of total brain homogenate samples and sarkosyl-pelleting fractions. Fig. S3. Development and validation of the in-house α-syn sandwich ELISA procedure. Fig. S4. Correlations of patients’ neuropathology scores with α-syn aggregation measurements. Table S1. Proteomic data summary of brain homogenates and sarkosyl-insoluble fractions. Materials and methods. [file 40035_2024_408_MOESM1_ESM.pdf]

SUPPLEMENTARY INFORMATION

Laferrière et al. *Biases in α-synuclein immuno-quantitation: a core problem for basic and ancillary studies of Parkinson's disease and multiple system atrophy.*

Supplementary figures and tables

Laferrière et al. - Figure S1

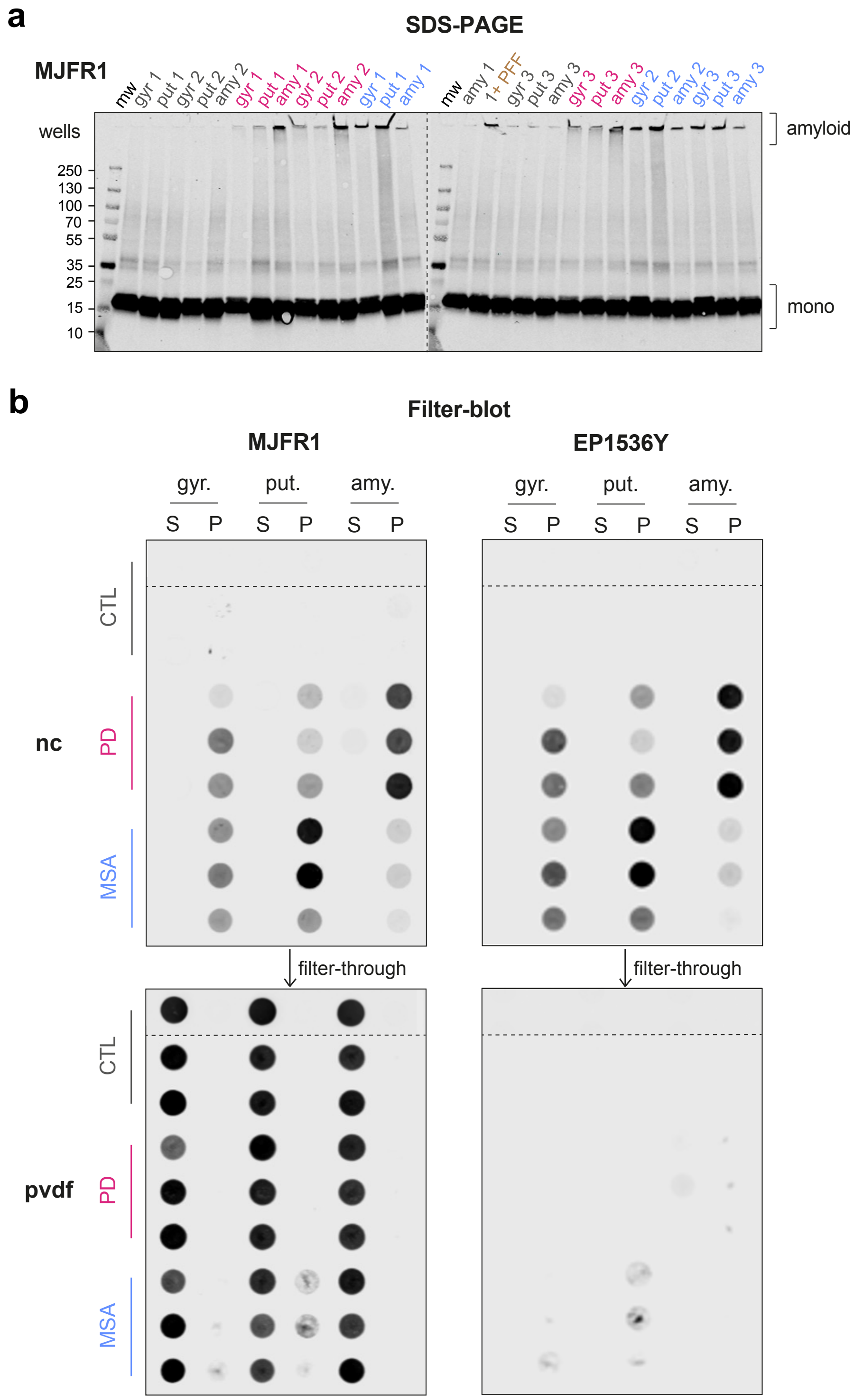

Fig. S1. SDS-PAGE and filter-blot of brain homogenate samples and sarkosyl-pelleting fractions.

a. SDS-PAGE analysis representative immunoblots of total α-syn (MJFR1) in the different brain samples from three controls (grey), PD (magenta) and MSA (cyan) cingulate gyrus, putamen and amygdala samples, corresponding to the picture shown in **Fig. 1a**. Stacking gels were kept and electro-transferred on the nitrocellulose membranes (wells). “mw”: molecular weight marker; “1+PFF”: control gyr 1 sample implemented with recombinant α-syn PFFs. **b.** Filter-blots of total (left, MJFR1) and pS129 phosphorylated α-syn (right, EP1536Y) retained on the top nitrocellulose (nc) and the underneath pvdf (filter-through) membranes in the sarkosyl pelleting supernatant (S) and pellet (P) fractions of the same samples, corresponding to the picture shown in **Fig. 1b** (dashed lines: separation of two membranes).

Laferrière et al. - Figure S2

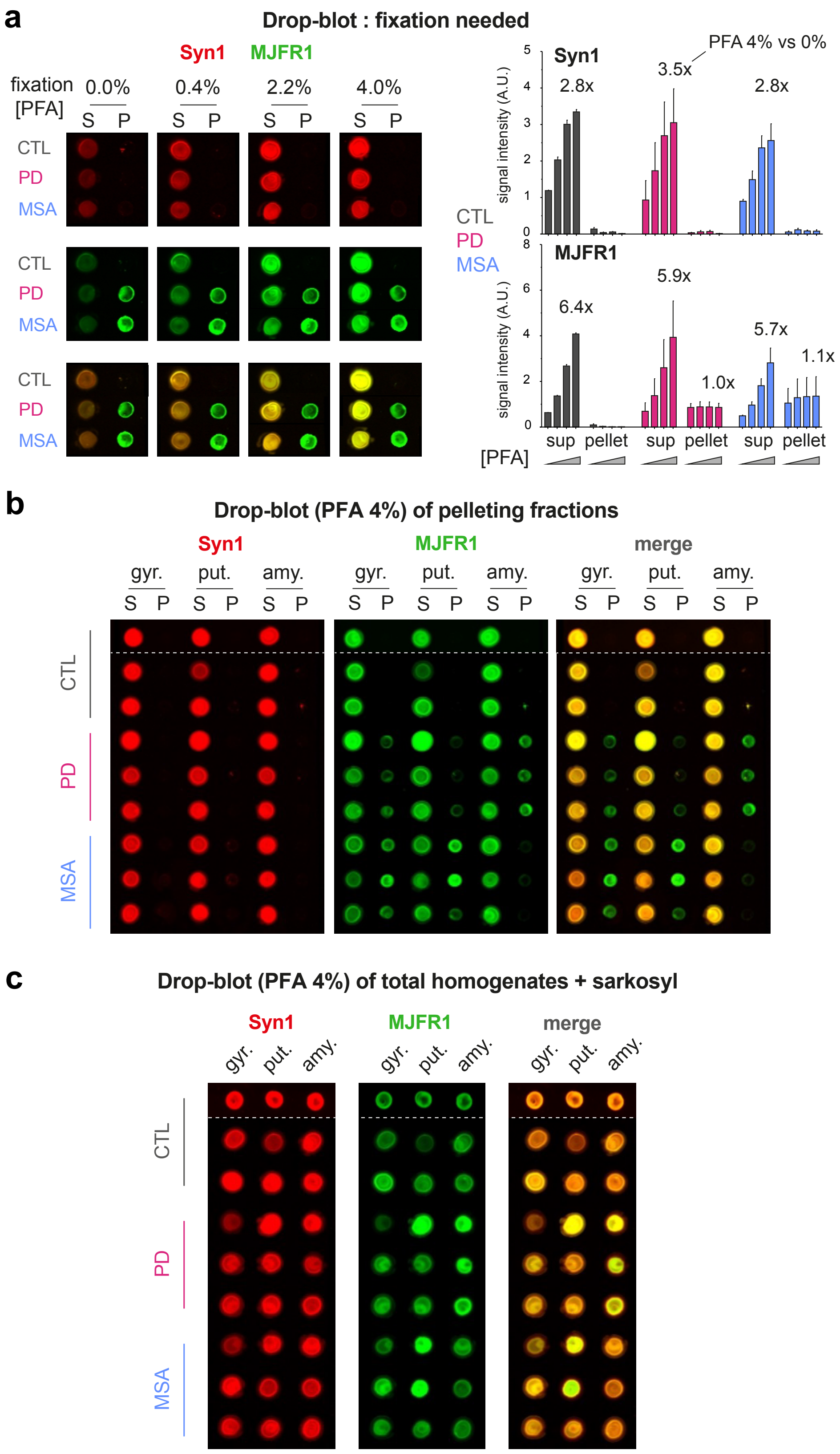

Fig. S2. Drop-blot of total brain homogenate samples and sarkosyl-pelleting fractions.

a. Drop-blot analysis of brain homogenates or pelleting fractions from three distinct brain regions of n=3 independent control, PD and MSA subjects respectively. Assessment of the effect of chemical PFA fixation on the immunoblot membrane after dropping the samples. Left: Drop-blots of non-amyloid (Syn1, red) and total (MJFR1, green) α-syn in the sarkosyl pelleting supernatant (S) and pellet (P) fractions according to PFA concentration in the fixation step. Right: Quantification of Syn1 (top) and MJFR1 (bottom) raw signal intensity (A.U.) in S and P fractions for 0 to 4% PFA post-drop fixation from three controls (grey), PD (magenta) and MSA (cyan) cingulate gyrus samples (mean +/-SD). PFA 4% vs 0% signal ratios are indicated above each positive signal detected. **b.** Drop-blots of non-amyloid (Syn1, red) and total (MJFR1, green) α-syn in pelleting fractions, corresponding to the picture shown in **Fig. 1c**. **c.** Drop-blots of non-amyloid (Syn1, red) and total (MJFR1, green) α-syn in total solubilized brain homogenates, corresponding to the picture shown in **Fig. 1d**. (dashed lines: separation of two membranes)

Laferrière et al. - Figure S3

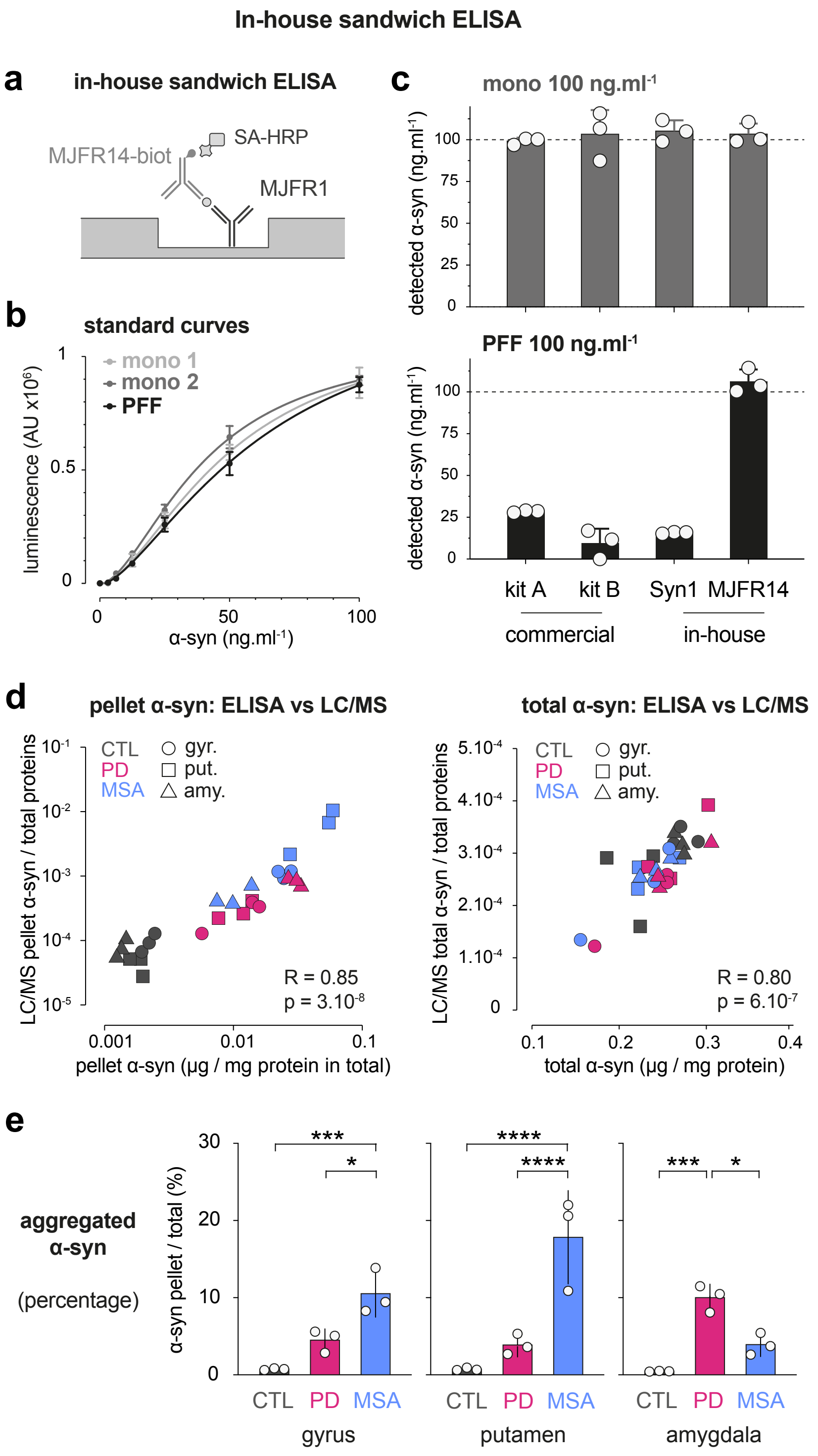

Fig. S3. Development and validation of the in-house α-syn sandwich ELISA procedure.

a. Schematic representation of our in-house sandwich ELISA set-up, with MJFR1 capture, biotinylated-MJFR14 detection and luminescence production with streptavidin-HRP and an ECL substrate. **b.** Standardization of the ELISA readout with serial dilutions of recombinant α-syn: mono 1: commercially available monomeric α-syn (Abcam); mono 2: in-house produced and purified monomers; and PFF: synthetic amyloids produced by fibrillization of mono 2 (mean +/-SD). **c.** Detected α-syn quantity in $ng.ml^{-1}$ by each of the indicated ELISA procedure (commercial kit A and kit B – see Material and Methods – or in-house ELISA with MJFR1 capture and Syn1 or MJFR14 detection) with an input of 100 $ng.ml^{-1}$ of α-syn monomers (top, grey) or PFF (bottom, black) (mean +/-SD). **c.** Individuals are plotted with in x-axis the α-syn amounts measured with our in-house ELISA in sarkosyl-insoluble pellets (left) or total brain homogenates (right) as µg of α-syn per mg of protein in total brain homogenates, and in y-axis the α-syn / total protein peptides abundance ratios measured by LC-MS on the insoluble pellet or total brain homogenates respectively. Pearson R correlations with their respective p values are indicated on the graph. Proteomic data are present in **Table S1**. **e.** The absolute quantifications of total and insoluble α-syn in **Fig. 1e** were used to calculate the precise percentage of aggregated α-syn among total α-syn population. These ratios are plotted as pellet / total α-syn for each of the samples (mean +/-SD). Significant differences obtained from Tuckey-corrected two-ways ANOVAs are represented (*p<0.05; **p<0.005; ***p<0.0005; ****p<0.0001).

Laferrière et al. - Figure S4

a **Neuropathology semi-quantitative scores**

Neuropathology scores (/5) :		α-syn inclusions			Neuronal loss		
**Type**	**ID#**	Cing. gyrus	Putamen	Amygdala	Cing. gyrus	Putamen	Amygdala
CTL	#1	0	0	0	0	0	0
CTL	#2	0	0	0	0	0	0
CTL	#3	0	0	0	0	0	0
PD	#1	3	0	2	1	0	3
PD	#2	3	0	3	3	1	3
PD	#3	2	1	5	2	0	3
MSA	#1	1	5	0	0	4	0
MSA	#2	2	5	0	0	4	0
MSA	#3	2	5	0	0	5	0

b **Correlation neuropathology scores / quantitations of α-syn species**

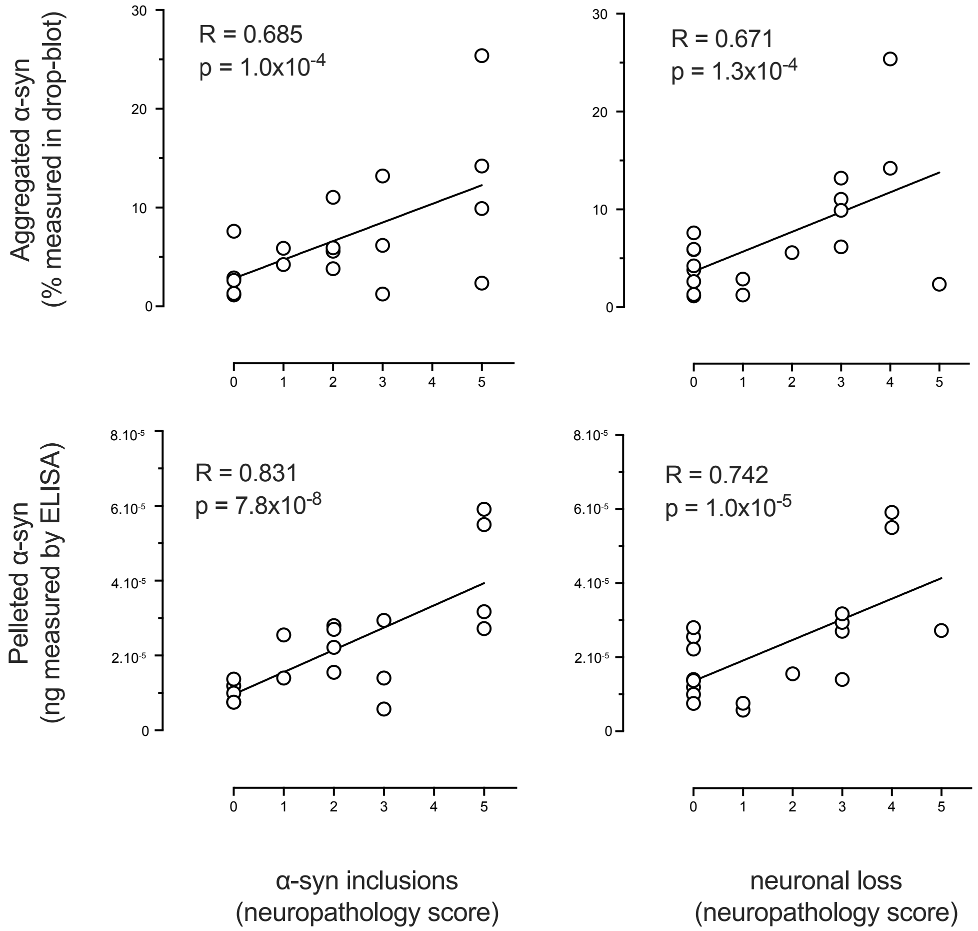

Fig. S4. Correlations of patients' neuropathology scores with α-syn aggregation measurements. **a.** Neuropathology semi-quantitative scores (/5) were obtained from the anatomopathology reports. The latter were performed by the brain bank neuropathologist through a microscopic examination of sections

(stained with pS129-α-syn for synucleinopathy inclusions and hematoxylin-eosin for neuronal loss scores respectively) of the respective brain regions on the other brain hemispheres. **b.** Individual plots of all PD and MSA cingulate gyrus, putamen and amygdala samples with in x axis the neuropathology scores (left: α-syn inclusions, right: neuronal loss) and in y axis the measurements performed in this study (top: % aggregated α-syn from drop-blots, bottom: pelleted α-syn amounts from ELISA). Linear regression and Pearson R correlations with their respective p values are indicated on the graphs.

Table S1. Proteomic data summary of brain homogenates and sarkosyl-insoluble fractions.

Detailed total protein and α-syn peptide counts obtained from the LC-MS analysis of total brain homogenates and sarkosyl-insoluble pellets used in **Fig. S3d**.

Samples		Brain homogenates		Sarkosyl-insoluble fractions	
**Region**	**Patient**	**all protein peptides**	**α-syn peptides**	**all protein peptides**	**α-syn peptides**
Gyrus	CTL 1	176 827 535 146	84 759 270	68 347 196 112	16 260 368
Gyrus	CTL 2	175 120 980 980	78 617 400	69 794 265 140	11 981 332
Gyrus	CTL 3	174 866 701 347	77 918 184	71 653 417 910	22 057 240
Gyrus	PD 1	176 162 529 557	29 793 565	66 181 806 065	22 263 261
Gyrus	PD 2	177 019 570 715	60 457 650	68 572 726 172	70 647 217
Gyrus	PD 3	177 152 458 025	61 627 457	68 171 689 988	60 933 456
Gyrus	MSA 1	175 154 968 929	33 074 853	68 630 003 225	175 821 759
Gyrus	MSA 2	175 877 012 518	70 565 854	69 253 593 209	209 282 296
Gyrus	MSA 3	171 374 189 236	59 506 277	66 384 291 347	196 731 162
Putamen	CTL 1	171 499 716 804	39 297 756	72 149 717 361	8 844 003
Putamen	CTL 2	173 828 759 303	70 808 407	66 357 985 457	4 825 994
Putamen	CTL 3	172 986 756 983	71 019 442	71 535 586 081	9 050 260
Putamen	PD 1	176 307 254 573	96 083 134	68 404 785 211	47 039 819
Putamen	PD 2	175 047 980 680	62 364 480	72 226 840 944	38 716 166
Putamen	PD 3	175 193 410 143	66 858 526	68 299 532 579	67 712 245
Putamen	MSA 1	171 672 338 945	67 029 467	66 485 877 335	1 183 701 812
Putamen	MSA 2	173 081 597 314	76 597 894	70 818 721 324	1 804 976 383
Putamen	MSA 3	172 450 421 813	57 384 387	68 832 608 209	373 941 500
Amygdala	CTL 1	173 084 576 543	77 060 006	68 038 888 934	13 013 965
Amygdala	CTL 2	175 474 147 853	72 645 815	66 140 022 760	9 757 705
Amygdala	CTL 3	170 369 614 696	81 683 914	69 162 857 109	17 608 494
Amygdala	PD 1	174 634 887 944	78 447 783	67 581 274 952	160 258 325
Amygdala	PD 2	173 827 199 304	60 010 932	64 534 698 649	138 795 574
Amygdala	PD 3	174 393 526 942	57 670 747	66 160 442 746	118 305 675
Amygdala	MSA 1	173 400 174 824	70 013 213	65 753 250 553	67 612 814
Amygdala	MSA 2	174 217 138 562	61 645 608	62 530 208 718	129 507 973
Amygdala	MSA 3	174 087 720 532	75 609 019	66 766 812 930	71 914 392

Material and methods

Human brain samples

Human subject anonymized information is provided below. Human samples from three different brain regions (cingulate gyrus, putamen and amygdala) were dissected from freshly frozen post-mortem brain samples from n=3 independent control, sporadic PD or MSA subjects respectively. Brain tissue samples were homogenized at 10% (w/v) in solubilization buffer (SB): 10 mM Tris pH 7.5, 100 mM NaCl, 0.1 mM EDTA, 1 mM DTT, Complete EDTA-free protease inhibitors (Roche) and PhosSTOP phosphatase inhibitors (Roche) using a gentleMACS Octo Dissociator (Miltenyi Biotec) with M Tubes, and the Protein extraction program. Protein concentration was determined using Pierce 660 nm Protein Assay kit (Thermo Fisher).

Human subject anonymized information

Type	ID#	DPM (hours)	Gender	Age at death	Diagnostic	Additional information	Brain regions
CTL	#1	10	M	73	Control without ND	Alzheimer Braak stage II	Gyr ; Put ; Amy
CTL	#2	30	M	85	Control without ND	Alzheimer Braak stage I	Gyr ; Put ; Amy
CTL	#3	21	F	80	Control without ND	N/A	Gyr ; Put ; Amy
PD	#1	31	M	75	Parkinson's disease	Transitionnal Lewy pathology + Alz Braak stage I	Gyr ; Put ; Amy
PD	#2	29	M	86	Parkinson's disease	Widespread Lewy pathology + Alz Braak stage III	Gyr ; Put ; Amy
PD	#3	36	F	62	Parkinson's disease	Widespread Lewy pathology	Gyr ; Put ; Amy
MSA	#1	48	F	78	Multiple system atrophy	MSA-type P	Gyr ; Put ; Amy
MSA	#2	22	M	75	Multiple system atrophy	MSA-type P	Gyr ; Put ; Amy
MSA	#3	39	M	72	Multiple system atrophy	MSA-type P	Gyr ; Put ; Amy

Sarkosyl-solubilization and pelleting procedure

For the extraction and purification of aggregates from brains samples, the pelleting procedure is similar to previously published protocols termed Sarkospin [6]. Samples were mixed 1:1 with SB 4% (w/v) N-lauroyl-sarcosine (sarkosyl, Sigma), 2 U.μl^{-1} Benzonase (Novagen) and 4 mM $MgCl_2$, reaching a final volume of 500 µl. Solubilization was then performed by incubating the samples at 37 °C under constant shaking at 600 rpm (Thermomixer, Eppendorf) for 45 min. Solubilized samples were then mixed 1:1 with SB 40% (w/v) sucrose, without sarkosyl, $MgCl_2$ or Benzonase, in 1 ml polycarbonate ultracentrifuge

tubes (Beckman Coulter) and centrifuged at 250,000 g for 1 hour at room temperature with a TLA 120.2 rotor using an Optima XP benchtop ultracentrifuge (Beckman Coulter). Supernatants were collected by pipetting. For filter-blot, drop-blot and SDS-PAGE immunoblot analysis, pellets were resuspended directly in the tube with 100 µL of the buffer corresponding to the supernatant (SB 1% sarkosyl 20% sucrose), and mixed with the same buffer in a fresh tube for reaching 1 ml (equal volumes to supernatant). For proteomics analysis, total solubilized homogenates were used directly, and sarkosyl-insoluble pellets were resuspended in 100 µL SB, and equalized for their total protein concentration quantified by Pierce 660 nm Protein Assay kit (Thermo Fisher), prior to denaturation in Laemmli buffer.

SDS-PAGE

For western blotting analysis of α-syn from brain samples, equal volumes of solubilized brain homogenates were added to Laemmli 1x final prior to denaturation at 95 °C for 5 min, and loaded on Mini-Protean TGX 4-20% gels (Biorad) followed by SDS-PAGE electrophoresis. Gels were transferred on nitrocellulose 0.2 µm membranes with Trans-Blot Turbo transfer system (Biorad) using the Mixed molecular weight program. Membranes were fixed, blocked and immunolabelled as described below.

Filter-blot

For filter-blot assays, 50 µl of native solubilized homogenates or supernatant/pellet fractions were filtered through layered nitrocellulose and PVDF 0.2 µm membranes (Protran, GE) using a dot blot vacuum device (Whatman). Membranes were fixed, blocked and immunolabelled as described below.

Drop-blot

For drop-blot assays, 2 µl of native solubilized homogenates or supernatant/pellet fractions were spotted onto nitrocellulose 0.2 µm membranes (Protran, GE) and air-dried. Membranes were fixed, blocked and immunolabelled as described below.

Immunolabelling

Western blot, filter-blot and drop-blot membranes were fixed for 30 min at room temperature in PBS with PFA (Sigma) at the indicated concentration, or 4% (v/v) final concentration if not specified. After three washes with PBS, membranes were saturated with 5% (w/v) skimmed powder milk in PBS-Tween20

0.05% (v/v) and probed with primary (overnight at 4 °C) and secondary (1 hour at room temperature) antibodies in PBS-T with 4% (w/v) BSA (see antibodies list) with three washes in PBS-T after each step. Immunoreactivity was measured by infrared using an Odyssey Scanner and Image Studio (Li-Cor).

Recombinant α-syn monomers and PFFs

Human monomeric recombinant α-syn was produced and purified as described in [3] for mono 2, or purchased from Abcam (ab254310) for mono 1. PFFs were obtained by agitation at 37 °C of the homemade monomeric α-syn as described in [3].

In-house sandwich α-syn ELISA

All incubation steps are performed on an orbital plate shaker at 400 rpm. Immuno Maxisorp C8 Lockwell White strips plates (Nunc) were coated overnight at 4 °C with 100 µl of 1 µg.ml^{-1} of the indicated capture antibody in 50 mM carbonate-bicarbonate coating buffer pH 9.2. Plates were washed three times with 250 µl PBS-Tween-20 0.05% (PBS-T), then blocked 2 hours at room temperature with 250 µl 1x casein blocking buffer (Sigma) prior to three more washes. Standards (recombinant monomeric or fibrillar α-syn) and samples are diluted in filtered Tris 20 mM, NaCl 100 mM, Tween 0.05%, BSA 0.5% and incubated in 100 µl final volume on plates at room temperature for 2 hours. Plates were washed three times with PBS-T and subsequently incubated with 100 µl of 1 µg.ml^{-1} of the indicated detection biotinylated antibody in 1x casein blocking buffer for 2 hours at room temperature. After three washes in PBS-T, plates were incubated with 100 µl of 1 µg.ml^{-1} of streptavidin-HRP in 1x casein blocking buffer for 1 hour at room temperature before three last washes in PBS-T. Chemiluminescence was measured by adding 100 µl of ECL Clarity or Clarity Max (BioRad) immediately before reading on a ClarioStar Plus reader (BMG LabTech).

Label-Free Quantitative LC-MS proteomics

Sample preparation and mass spectrometry analysis were performed as previously described [6]. Briefly, protein samples were concentrated and cleaned using a preparative short SDS-PAGE. Bands were cut and destained before overnight trypsin digestion at 37 °C. Peptides were extracted, dried and resuspended in 0.1% HCOOH before LC-MS/MS analysis on an Ultimate 3000 nanoLC system (Dionex) coupled to an Electrospray Orbitrap Fusion Lumos Tribrid Mass Spectrometer (Thermo Fisher). Data

were searched by SEQUEST through Proteome Discoverer 2.5 (Thermo Fisher) against the Homo sapiens Reference Proteome Set. Peaks were detected and integrated using the Minora algorithm embedded in Proteome Discoverer. Proteins were quantified based on unique peptides intensities. Normalization was performed based on total protein amount. The mass spectrometry proteomics data have been deposited to the ProteomeXchange Consortium via the PRIDE partner repository with the dataset identifiers **PXD039575**.

Antibodies used in this study

Name	Target (theory)	Company	Cat.No	Dilution
**Primary antibodies**				
MJFR-1	human alpha-synuclein	Abcam	ab138501	1 : 10,000
Syn1	alpha-synuclein	BD	610787	1 : 2,000
EP1536Y	pS129 phospho-synuclein	Abcam	ab51253	1 : 5,000
Actin	beta-actin	Sigma	A5316	1 : 10,000
**Secondary antibodies**				
Goat anti-mouse IRDye 680RD	mouse IgG (H+L)	LI-COR	926-68070	1 : 5,000
Goat anti-rabbit IRDye 800CW	rabbit IgG (H+L)	LI-COR	926-32211	1 : 5,000
**ELISA capture antibodies**				
MJFR-1	human alpha-synuclein	Abcam	ab138501	1 µg/ml
Syn1	alpha-synuclein	BD	610787	1 µg/ml
Syn2	alpha-synuclein	Bio Legend	#848302	1 µg/ml
D37A6	rodent alpha-synuclein	Cell signalling	#4179	1 µg/ml
EP1536Y	pS129 phospho-synuclein	Abcam	ab51253	2 µg/ml
**ELISA detection antibodies**				
MJFR-14-6-4-2 (biotin)	alpha-synuclein aggregate	Abcam	ab227047	0.5 µg/ml
LB509 (biotin)	human alpha-synuclein	Bio Legend	#807710	0.5 µg/ml
Syn2 (biotin)	alpha-synuclein	Bio Legend	#848306	0.5 µg/ml
D37A6 (biotin)	rodent alpha-synuclein	Cell signalling	#74184	0.5 µg/ml
Streptavidin-HRP		Jackson IR	016-030-084	0.5 µg/ml
**Commercial alpha-synuclein ELISA kits**				
Kit A	human alpha-synuclein	Abcam	ab260052	
Kit B	human alpha-synuclein	Thermo	#KHB0061	

Chemicals and kits used in this study

Name	Company	Cat.No
**Chemicals and kits**		
Complete EDTA-free protease inhibitors	Roche	11873580001
PhosSTOP phosphatase inhibitors	Roche	4906845001
Pierce 660nm Protein assay kit	Thermo Fisher	22660
N-lauroyl-sarcosine (sarkosyl)	Sigma	61743
Benzonase nuclease	Novagen	70746-4
PFA (37% v/v in MetOH 10% v/v)	Sigma	252549
Tween-20	Sigma	P9416-100ML
Bovine Serum Albumin	Sigma	810533
SDS-PAGE Protean TGX Gel migration	Biorad	4561045
SDS-PAGE Transfer Transblot Turbo	Biorad	1704159
Nitrocellulose 0.2 membrane	GE Amersham	10600001
PVDF 0.2 membrane	GE Amersham	10600021
Casein Blocking buffer (10x)	Sigma	B6429
ECL Clarity	Biorad	1705061
ECL Clarity Max	Biorad	1705062

Statistics

Statistical analyses were all performed using GraphPad Prism 9. Immunoblotting signal intensity and proteomic analysis were also done and plotted using the software GraphPad Prism 9. Comparisons on **Fig. 1d-e**, and **Fig. S3e** were made using two-ways ANOVAs with Tukey's correction for multiple comparisons. Significant differences are represented with their adjusted p values as: *p<0.05; **p<0.005; ***p<0.0005; ****p<0.0001. Two-tailed Pearson R correlations were used to correlate the different variables obtained from single individuals on **Fig. S3d** and **Fig. S4b**. Simple linear regression were used between neuropathology scores and biochemical assays measurements on **Fig. S4b**.
